# Case of Urinary Calculus with Congenital Malformation of the Penis

**Published:** 1847-11

**Authors:** George D. Stevens

**Affiliations:** Williamsville


ART. II.—*Case of Urinary Calculus with congenital malformation of the Penis.* By George D. Stevens, M. D.

On the morning of the 2nd of August, I was called to visit a son of Jacob Rummell, aged 8 years. On my way to his residence, I learned the following particulars regarding the case:—The morning previous to my visit, the lad betrayed symptoms of uneasiness, and and on being interrogated, told his parents he could not urinate; after witnessing several unsuccessful attempts, the mother prepared some pumpkin-seed tea, which she gave him freely, with other diluent drinks of various kinds, all of which proved ineffectual. Had never been affected so before, but the father said he had noticed that his son always projected a very small stream when he urinated, and that his penis "was not quite right."

I found the hypogastrium tensely distended, tender to the touch, the patient suffering much, and determined to cathererize immediately. On searching for the *meatus urinarius*, found an indentation resembling it, but imperforate; but the search revealed a very small opening beneath a fold of the *frænum preputii* which proved to be the commencement of the urethra; and was too minute to admit a small sized catheter. After dilating it with a probe-pointed bistoury, the catheter passed easily until it reached the neck of the bladder, which offered some resistance, but by moderately continued pressure it passed. The bladder was soon emptied of about a pint and a half of urine, presenting no unusual appearance, but strongly ammoniacal. Regarding the case as one of spasm of the neck of the bladder I directed a cathartic of ol. Ricini and spts. Terebinth. fomentations to hypogastrium of laudanum and spts. of Camphor.

August 3rd, 5 o'clock P. M. Found patient with bladder distended, betraying symptoms of increased suffering; removed about the same quantity of urine as before, possessing the same characteristic odor; patient quite easy. I wished to leave the instrument in the bladder, but when the patient was left alone for a few moments, he would immediately withdraw it, so far as to render it useless. The prescriptions of the day before not having been carried out, I directed the parents to employ them thoroughly, and, at the same time, to administer enemas, as he had passed some ascarides. He was also to take a powder of camphor and opium every four hours spts. nit dulc, at intervals between the time of taking the powders. Directed the Father to call me next day if these remedies did not succeed. Was not called on the 4th, and began to feel assured of my patient's recovery.

On the morning of the 5th, a messenger came requesting my speedy attendance: saw patient at 10 A. M., presenting appearances similar to those on my former visits. I had not been called the day before "because he was so comfortable, it was thought unnecessary." Passed the catheter, urine flowed freely until half a pint was discharged, when it suddenly ceased; at the same time a sensation of grasping was communicated to the instrument, and the boy cried out with extreme pain. Withdrew the catheter, and found no obstruction in it. As the arrest of the flow of urine seemed so much like the effect of violent spasm, I determined to rest for half an hour, and at the end of that time, passed the instrument as I thought fully into the bladder, no urine flowed; in a short time the same sensation as before was communicated to the catheter, the patient expressing intense pain, and I began to think that a stone might produce the difficulty. Placed the patient upon his back with his hips raised, left with intention of visiting him again in the evening, urgent symptoms being allayed for a time. In the evening Dr. Ham saw the patient with me: the patient had passed some urine, still the hypogastric region was full, and tender to touch. On attempting to introduce the catheter, an obstruction was discovered beneath the arch of the pubis; a sound was tried which rendered it certain that a foreign body was lodged in the tract of the urethra; it appeared hard and somewhat rough; we came to the conclusion that it was a calculus, as the aperture of the urethra had been too small to admit the introduction of any substance by the patient previous to its being dilated. After dilating the urethra as much as was consistent, administering a cathartic of calomel and bitartras of potassa, we left with a slight hope of its being discharged, if not, expecting on the following morning to cut upon and remove it. Mr. R., however, came early, bringing with him a calculus about the size of a white bean, spheroidal in form, surface rough, color brown. When broken it seemed made up of concentric layers, which were distinct, encompassing a nucleus of a grayish color, and much harder than the external layers. When dry it weighed six grains.

This case is chiefly interesting, from the length of time the calculus must have existed before any symptoms of its presence manifested themselves, from the structure of the *meatus urinarius* (which latter stands perhaps in the relation of cause to the effect) and from the size of the calculus passed by a lad of eight years.

WILLIAMSVILLE, September 15, 1847.

